# Transcriptomic Analysis of the Mouse Mammary Gland Reveals New Insights for the Role of Serotonin in Lactation

**DOI:** 10.1371/journal.pone.0140425

**Published:** 2015-10-15

**Authors:** Jimena Laporta, Francisco Peñagaricano, Laura L. Hernandez

**Affiliations:** 1 Department of Animal Sciences, University of Florida, Gainesville, Florida, United States of America; 2 University of Florida Genetics Institute, University of Florida, Gainesville, Florida, United States of America; 3 Department of Dairy Science, University of Wisconsin, Madison, Wisconsin, United States of America; Wayne State University, UNITED STATES

## Abstract

Serotonin regulates numerous processes in the mammary gland. Our objective was to discover novel genes, pathways and functions which serotonin modulates during lactation. The rate limiting enzyme in the synthesis of non-neuronal serotonin is tryptophan-hydroxylase (TPH1). Therefore, we used TPH1 deficient dams (KO; serotonin deficient, *n* = 4) and compared them to wild-type (WT; *n* = 4) and rescue (RC; KO + 100 mg/kg 5-hydroxytryptophan injected daily, *n* = 4) dams. Mammary tissues were collected on day 10 of lactation. Total RNA extraction, amplification, library preparation and sequencing were performed following the Illumina mRNA-Seq. Overall, 97 and 204 genes (false discovery rate, FDR ≤ 0.01) exhibited a minimum of a 2-fold expression difference between WT *vs*. KO and WT *vs*. RC dams, respectively. Most differentially expressed genes were related to calcium homeostasis, apoptosis regulation, cell cycle, cell differentiation and proliferation, and the immune response. Additionally, gene set enrichment analysis using Gene Ontology and Medical Subject Headings databases revealed the alteration of several biological processes (FDR ≤ 0.01) including fat cell differentiation and lipid metabolism, regulation of extracellular signal-related kinase and mitogen-activated kinase cascades, insulin resistance, nuclear transport, membrane potential regulation, and calcium release from the endoplasmic reticulum into the cytosol. The majority of the biological processes and pathways altered in the KO dams are central for mammary gland homeostasis. Increasing peripheral serotonin in the RC dams affects specific pathways that favor lactation. Our data confirms the importance of serotonin during lactation in the mammary gland.

## Introduction

Serotonin (5-hydroxytryptamine, 5-HT) is a monoamine that is biochemically derived from the essential amino acid L-tryptophan. Tryptophan hydroxylase (TPH) is the rate-limiting enzyme in serotonin synthesis and converts L-tryptophan into 5-hydroxy-L-tryptophan (5-HTP) which is then converted to serotonin. The expression of two independent TPH enzymes, codified by different genes, results in two serotonergic systems: a neuronal, with TPH2 as the rate-limiting enzyme, and a non-neuronal, with TPH1 as the rate-limiting enzyme [1,2]. Serotonin is largely known for its role as a neurotransmitter in the CNS, where 2% of total serotonin is produced. Serotonin within the CNS influences a variety of behavioral functions, including circadian rhythms, maternal behavior, sleep-wake cycle, appetite, aggression, sexual behavior, sensorimotor reactivity, pain sensitivity, memory, and learning, among others [3,4]. However, there is increasing evidence supporting the role of non-neuronal (peripheral) serotonin, which accounts for 98% of total serotonin in the body, in physiological functions and metabolic processes [5,6]. In addition, peripheral serotonin acts through more than 15 different receptors that possess unique spatiotemporal distribution [7]. This enables the possibility for multiple, simultaneous physiological outcomes that can be attributed to serotonin across and within tissues.

In the mammary gland, a complete serotonergic network exists and acts in an autocrine-paracrine manner regulating processes such as calcium homeostasis [8,9], tight junction permeability [10,11], milk protein gene expression [12,13] and mammary gland homeostasis during lactation [14]. The *Tph1* deficient model has been used to address the participation of specific candidate pathways in the mammary gland, bone and gut [5,8,15,16,]; however, changes in genome-wide expression have not been addressed in the mammary gland of this mouse model.

Given the participation of serotonin in several important physiological functions necessary for lactation, we hypothesized that disruption of serotonin’s rate limiting enzyme TPH1 will impact the transcriptome of the lactating mammary gland. Therefore, the objective of this study was to investigate the transcriptome of the lactating mammary gland using a global *Tph1* deficient mouse model to discover novel genes, pathways and processes that might be altered by the absence of serotonin. Additionally, rescuing the KO phenotype by exogenously administering 5-HTP, will enable the discovery of novel genes affected by serotonin, as 5-HTP is a selective precursor for serotonin synthesis. Discovering new roles and functions for serotonin in the mammary gland can provide opportunities for manipulation of this pathway to improve lactation performance and efficiency.

## Materials and Methods

### Mammary tissue collection and RNA extraction

Mammary gland samples used in this experiment were collected from a previous study [9]. All experiments were performed under protocols approved by the Research Animal Care and Use Committee at the University of Wisconsin-Madison (#A1473). Briefly, twelve pregnant female C57B6/J mice were housed individually and maintained in a controlled environmental facility for biological research in the Department of Animal Sciences, University of Wisconsin-Madison, at a temperature of 25°C, 50–60% humidity, and a 12-h light/dark with free access to food and water. Pregnant dams (day 15 of gestation) were assigned to 1 of 3 groups: group 1 consisted of *Tph1* deficient dams (KO, *n* = 4), group 2 consisted of *Tph1*-KO mice injected daily (i.p.) with 100 mg/kg of 5-HTP (rescue, RC, *n* = 4) to rescue peripheral serotonin function, and group 3 consisted of wild-type dams (WT, *n* = 4). The WT and KO dams were injected daily with saline to control for the variable of stress due to injection. Injections began on day 15 of gestation and ended on day 10 of lactation. Dams were euthanized (CO_2_ and decapitation) on the morning of day 10 of lactation and mammary glands were collected. Specifically, the entire mammary gland was removed and a visually homogeneous piece of tissue was selected for RNA extraction, snap frozen in liquid nitrogen, and stored at -80°C until used. The epithelial tissue was not separated from the surrounding stroma. Total RNA was isolated from mammary gland tissue following the TRI-Reagent (Molecular Research) protocol. The quality and concentration of the extracted RNA was assessed using Agilent Bioanalyzer chips (RIN = 7.1 ± 1.1). The average circulating serotonin concentrations were 142 ± 31 ng/mL for the WT group, 19.5 ± 9 ng/mL for the KO group, and 284 ± 26 ng/mL for the RC group [9].

### Library generation and RNA sequencing

Libraries from total RNA from individual samples were prepared following the standard procedures for the Illumina’s mRNA-Seq. Sequencing libraries were prepared from 50 ng RNA per sample and sequenced with Illumina’s HiSeq 2000 at the University of Wisconsin-Madison Biotechnology Center. The 12 libraries were barcoded, multiplexed, and sequenced in two HiSeq 2000 lanes. A read was defined as a 100 bp cDNA fragment sequenced from a single end.

### Mapping reads to the reference genome

Sequence reads were mapped to the reference mouse genome (GRCm38) using the software package Tophat (v2.0.13) [17, 18]. Two rounds of alignments were performed in order to maximize sensitivity to splice junction discovery: first, novel splice junctions were discovered in each sample independently, and then these novel splice junctions plus known splice junctions from the ENSEMBL annotation were combined and supplied to Tophat for a second alignment. This two-step approach allows for full utilization of the novel junctions identified in each of the samples. Read alignments having more than 2 mismatches or mapped equally well to more than 40 genomic locations were discarded.

### Assembly of transcripts and normalization of the RNA-Seq data

The resulting alignments were used to reconstruct transcript models using the software package Cufflinks (v2.2.1) [19]. In addition, the tool cuffmerge was used for merging each of the assemblies with the reference mouse annotation file in order to combine novel transcripts with known annotated transcripts. This strategy maximizes the quality of the final assembly. Transcript abundances were scaled via the median of the geometric means of fragment counts across all libraries [20] and corrected for multiple-mapping reads and sequence bias [21] in order to improve expression estimates.

### Identification of differentially expressed genes and gene set enrichment analysis

Differentially expressed genes (DEG) were detected using Cuffdiff [22]. This software controls for cross-replicate variability taking into account read-mapping ambiguity. This is achieved by using a model for fragment counts based on the beta negative binomial distribution [22]. The analyses were performed using the default settings for all parameters. *P*-values reported by Cuffdiff were corrected for multiple testing using the Benjamini-Hochberg procedure [23]. The significant enrichment of Gene Ontology (GO) and Medical Subject Headings (MeSH) terms with genes differentially expressed between treatments was analyzed using Fisher’s exact test, a test of proportions based on the cumulative hypergeometric distribution [24]. For each comparison of interest, genes that showed a FDR ≤ 0.10 and had ENSEMBL annotations were tested against the background set of all expressed genes with ENSEMBL annotations. The GO and MeSH enrichment analyses were performed using *goseq* package [25] and *meshr* package [26, 27], respectively, which are available in the R environment [28]. Functional categories with a FDR ≤ 0.01 were considered significant.

### Validation of RNA-Seq results by assessment of gene expression by real-time PCR

Four differentially expressed genes were chosen for validation of RNA-Seq results: period circadian clock 2 (Per2), G protein-coupled receptor 113 (Gpr113), GLIS family zinc finger 3 (Glis3), and one novel transcript unit (NTU, located in chr5:30,208,007–30,212,059). The validation was performed using quantitative real-time PCR (qPCR) conducted with the CFX96 Touch Real-Time PCR Detection System (Bio-Rad). A total of 1 μg RNA from each sample was used to synthesize cDNA using the iScript cDNA synthesis kit (Bio-Rad Laboratories, CA) and diluted 1:5 in dH_2_O. Reaction mixtures and cycling conditions were performed as previously described [9]. A positive and negative (non-template control, NTC) control was added to the PCR plate. Each sample was assessed in duplicate and the %CV between the duplicates was < 2%. All primers sequences for the validated genes were designed to span exon-exon junctions to minimize the potential of amplifying genomic DNA (S1 Table). Ribosomal protein S15 (Rps15) was used as the housekeeping gene and the relative gene expression was calculating using the method 2^-ΔΔCt^ with the WT group as the control group [29].

## Results

### Exploration and evaluation of overall gene expression

Genome-wide gene expression in mammary gland samples was evaluated using RNA-Seq technology. These samples belong to WT dams, with normal circulating serotonin concentrations, *Tph1* KO dams with minimal circulating serotonin concentrations, and RC dams with elevated circulating serotonin concentrations. A summary of sequencing read alignments to the reference genome is presented in S2 Table. Illumina sequencing was effective at producing large numbers of high quality reads from all samples. On average, 79% of the total reads were mapped successfully. Among the aligned reads, 97% were mapped to unique genomic regions. Sequencing data can be accessed by GEO with the accession number GSE68315.

**Fig 1:**
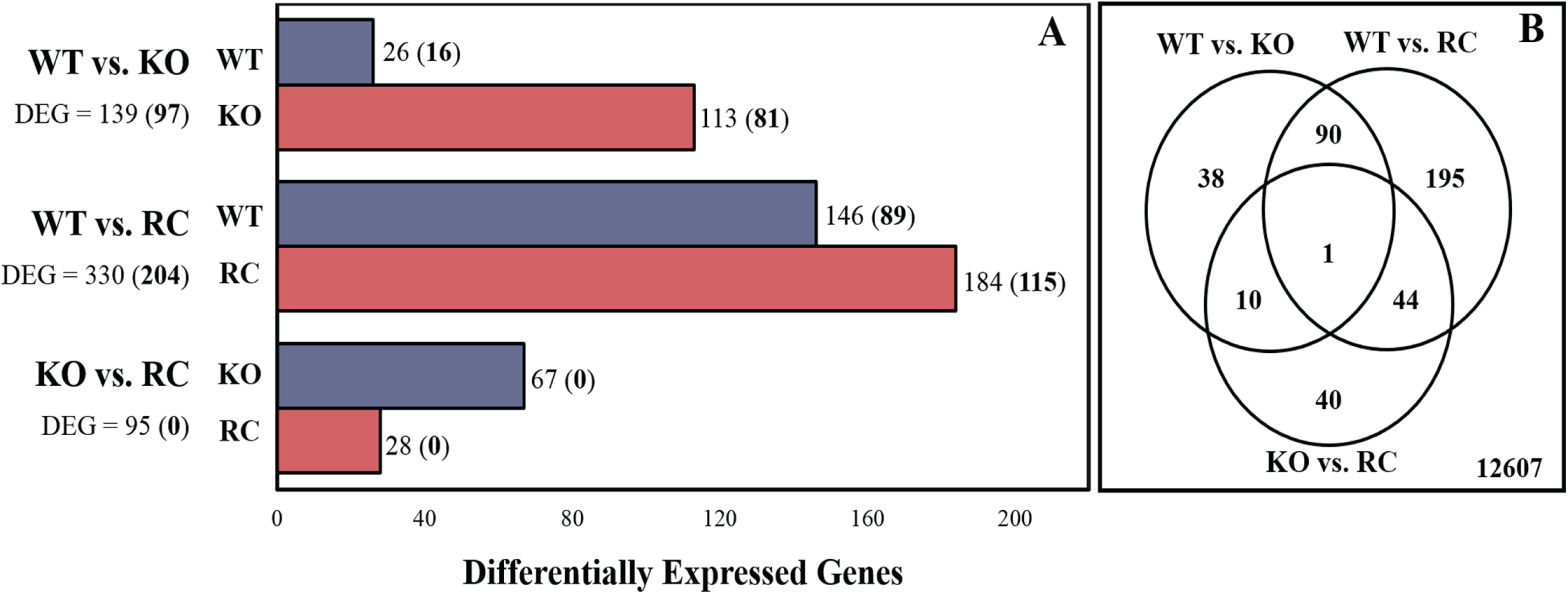
Comparison of overall gene expression between the three treatment groups. Mammary gland samples belong to wild-type dams (WT), Tryptophan hydroxylase (*Tph1*) deficient dams (serotonin deficient; KO), and *Tph1* deficient dams injected daily with 5-hydroxytryptophan (RC). (**A**) The bar graph shows the number of genes upregulated in each group for each of the three pairwise comparisons. The numbers represent the total number of differentially expressed genes (DEG) at 5% FDR (and at 1% FDR in parenthesis). (**B**) The Venn diagram shows the overlap between genes that showed significant differential expression at 5% FDR in each of the three pairwise comparisons.

For the WT *vs*. KO pairwise comparison, a total of 97 genes were differentially expressed (FDR ≤ 0.01 and fold change ≥ 2). Of the DEG, 81 were upregulated and 16 were downregulated in the KO dams (Fig 1A). For the WT *vs*. RC pairwise comparison, a total of 204 genes were differentially expressed (FDR ≤ 0.01 and fold change ≥ 2). Of the differentially expressed genes, 115 genes were upregulated and 89 were downregulated in the RC compared to the WT (Fig 1A). For the KO *vs*. RC pairwise comparison, there were no DEG at a FDR of 1%; however controlling FDR at 5%, 95 genes showed differential expression, and of those 67 were upregulated and 28 were downregulated in the mammary gland of RC dams. The overlap between genes that showed differential expression at a 5% FDR in each of the three pairwise comparisons can be seen in the Venn diagram in Fig 1B. Note that 90 significant genes were detected simultaneously between WT *vs*. KO dams, and between WT *vs*. RC dams. The full list of differentially expressed genes (FDR ≤ 0.01 and fold change ≥ 2) detected in each pairwise comparisons can be found in S1 and S2 Files, respectively.

### Characterization of differentially expressed genes

In an effort to identify functions and processes related to serotonin that might be relevant to lactation and mammary physiology, the DEG were characterized and classified using the annotation provided by different databases including Gene Ontology, WIKI pathways and KEGG pathways. Only the most relevant functions and processes were selected, and only terms with 3 or more significant genes are displayed.

Out of the 90 significant genes detected simultaneously in the two pairwise comparisons involving the WT mammary glands (Fig 1B), 62 showed a fold change ≥ 2 and FDR ≤ 0.01. Table 1 displays the functional characterization for these 62 significant genes. It is important to note that both the KO and RC dams do not possess a functional Tph1 protein. Notably, several of the DEG belonged to immune related functions such as interleukin (IL) -6, tumor necrosis factors (TNF) and fragment crystallizable (Fc, found on the surface of immune cells) signaling pathways, and the majority of these genes were upregulated in the mammary gland of KO and RC dams compared to WT dams. Furthermore, functions such as cell proliferation, and different signal transduction mechanisms (e.g., MAP kinase and small GTPases) and pathways (e.g., epidermal and transforming growth factors, EGF, and TGF-β) were also identified. Only a few genes were downregulated simultaneously in KO and RC dams compared to the WT dams [e.g., 3-hydroxy-3methylglutaryl-Coenzyme A synthase 1 (Hmgcs1); purinergic receptor P2X, ligand-gated ion channel (P2rx7); ect2 oncogene (Ect2); and interleukin 2 receptor, alpha chain (Il2ra)].

**Table 1:** Functional characterization of the 62 most significant genes (FDR ≤ 0.01 and fold change ≥ 2) detected simultaneously in the mouse mammary gland on day 10 of lactation between WT *vs*. KO dams and WT *vs*. RC dams.

Functional Characterization	Differentially expressed genes
**Cellular response to calcium ion**	Fosb, Jun, Ect2
**Cell cycle**	Sik1, Dusp1, Mapk4, Ect2
**Cell differentiation**	Klf4, Sik1, Ect2
**Signal transduction**	Gem, Akap12, Socs3, Gpr113, Ect2
**Regulation of cell proliferation**	Klf4, Ptgs2, Serpine1, Atf3, Jun, Zbtb16
**Small GTPase signaling**	Rab30, Gem, Rhod
**MAP kinase signaling pathway**	Nr4a1, Dusp1, Mapk4, Jun, Hspa1a
**EGFR1 signaling pathway**	Dusp, Jun, Socs3
**TGF-b signaling pathway**	Fosb, Atf3, Jun
**Protein phosphorylation**	Sgk1, Sik1, Mapk4, Trib1, P2rx7
**Insulin signaling**	Sgk1, Mapk4, Jun, Socs3
**Inflammatory response**	Ier3, Zfp36, Socs3, Il2ra
**Immune system**	Nr4a1, Hbegf, Jun, Socs3, Zbtb16, Il2ra, P2rx7
**IL-6 signaling pathway**	Sgk1, Jun, Socs3
**TNF signaling pathway**	Ptgs2, Jun, Socs3
**Fc signaling pathway**	Nr4a1, Hbegf, Jun
**Metabolism**	Got1, Ptgs2, Pygm, Hmgcs1
**Angiogenesis**	Hbegf, Ptgs2, Jun
**Fibroblast proliferation**	Fosl2, Serpine1, Jun

WT = wild-type mice; KO = Tryptophan hydroxylase (*Tph1*) deficient mice; RC = Rescue mice (*Tph1* KO + 100 mg/kg daily injections of 5-hydroxytryptophan). Gene symbols that are underlined denote genes downregulated in the mammary gland of KO and RC dams compared to WT dams.

Next, we characterized the 97 DEG (FDR ≤ 0.01 and fold change ≥ 2) detected in the pairwise comparison between the WT and KO dams. Table 2 shows the functions and processes in which these DEG are involved. Lack of peripheral serotonin impacted processes and functions such as apoptosis, adipogenesis, insulin signaling, glucose metabolism and angiogenesis (Table 2). Notably, all the DEG associated with these functions were upregulated in the mammary gland of KO dams compared to the WT dams.

**Table 2:** Functional characterization of the 97 most significant genes (FDR ≤ 0.01 and fold change ≥ 2) detected in the mouse mammary gland on day 10 of lactation between WT *vs*. KO dams. These are functional terms that were detected exclusively in this specific comparison.

Functional Characterization	Differentially expressed genes
**Apoptotic process**	Ece1, Sgk1, Nr4a1
**Negative regulation of ERK**_**1/2**_	Klf4, Ndrg2, Dusp1
**Signaling by GPCR**	Cxcl1, Hbegf, Ect2
**Angiogenesis**	Ptgs2, Jun, Epgn, Hbegf
**Inflammatory response**	Ptgs2, Cxcl1, P2rx7
**Immune system**	Jun, zbtb16, P2rx7, Il2ra
**Glucose metabolism**	Dusp1, Pygm, Got1
**Insulin signaling**	Jun, Sgk1, Dusp1
**Adipogenesis**	Serpine1, Egr2, Socs3
**Serotonergic synapse**	Ptgs2, Dusp1, Tph1

WT = wild-type mice; KO = Tryptophan hydroxylase (*Tph1*) deficient mice. Gene symbols that are underlined denote genes downregulated in the mammary gland of KO dams compared to WT dams.

We characterized also the most important functions for the 204 DEG (FDR ≤ 0.01 and fold change ≥ 2) detected in the pairwise comparison between WT and RC dams (Table 3). Notably, exogenous administration of 5-HTP impacted several functions in the mammary gland. Twelve DEG were associated with lipid metabolic processes: 11 out of 12 were upregulated in the RC dams compared to the WT dams. In addition, functions associated with oxidation-reduction processes were also affected, in this case only 50% of the DEG were upregulated in RC dams compared to the WT dams. Physiological functions associated with mammary gland cell remodeling and renewal, including cell migration, cell adhesion, cell chemotaxis, senescence and autophagy, cell death and cell growth, were also affected by the increased circulating serotonin in the RC group. The majority of the DEG associated with these functions were upregulated in the mammary gland of RC dams compared to the WT dams. Interestingly, the DEG associated with cell division were all downregulated in RC dams (e.g., Ect2, Sept5, Kif20b, and Kif11).

**Table 3:** Functional characterization of the 204 most significant genes (FDR ≤ 0.01 and fold change ≥ 2) detected in the mouse mammary gland on day 10 of lactation between WT *vs*. RC dams. These are functional terms that were detected exclusively in this specific comparison.

Functional Characterization	Differentially expressed genes (WT *vs*. RC)
**Lipid metabolic processes**	Ptgs2, Agps, Pla2g4f, Plch2, Insig1, Ept1, Thrsp, Acsl6, Acly, Hmgcs1, lipin1, Apod
**Oxidation-reduction process**	Ptgs2, Cyp4b1, Dhodh, Pyroxd2, Agps, Alox5ap, Gsr, Nqo1
**Extracellular matrix organization**	Serpine1, Mmp3 **,** Adamts5
**Cell division**	Ect2, Sept5 **,** Kif20b, Kif11
**Cell migration**	Nfatc2, Flt1, Spata13, Hbegf
**Cell adhesion**	Mybpc2, Cdhr1, Gp1bb
**Cell chemotaxis**	Kit, Plxnb3, Ccl28, Flt1
**Senescence and autophagy**	Jun, Serpine1, Igfbp5 **,** Mmp3
**Positive regulation of cell death**	Ptgs2, Hp, Egr1
**Positive regulation of cell growth**	Slc25a33, Sgk1, Hbegf
**Positive regulation of nitric oxide biosynthesis**	Ptgs2, Klf4, Agt
**Positive regulation of cAMP biosynthesis**	Pthlh, Akap12, Crhr1
**Positive regulation of cytosolic calcium concentration**	Chrna9, Crhr1, Ccl28
**T cell differentiation**	Cacnb4, Egr1, Kit
**IL-5 signaling pathway**	Jun, Pim1, Alox5ap
**Cytokine signaling in immune system**	Socs3, Flt1, Ccl28, Il2ra, Usp18, Il13ra1, Kit
**Interferon signaling**	Socs3, Ifit1 **,** Usp18
**SLC-mediated transmembrane transport**	Slc29a3, Slc22a1, Slc41a2, Slc5a8
**Metabolism of amino acids**	Glul, Got1, Nqo1
**GPCR ligand binding and Gα(i) signaling events**	Crhr1, Cck, Agt, Drd4, Pthlh, Ccl28
**Regulation and signaling by Rho GTPases**	Spata13, Srgap3, Rhod, Arhgef37
**Ras signaling pathway**	Pla2g4f, Flt1, Efna5 **,** Kit
**Signaling by PDGF**	Ect2, Srgap3, Rhod
**Signaling by EGFR**	Nr4a1, Hbegf, Kit
**Peroxisome**	Acsl6, Abcd2, Agps
**Tight junction**	Mylpf, Myh4, Actn3
**Endocytosis**	Flt1, Hspa1a, Il2ra, Kit
**Calcium ion transport**	Cacnb4, P2rx7, Atp2b1

WT = wild-type mice; RC = Rescue mice (*Tph1* KO + 100 mg/kg daily injections of 5-hydroxytryptophan). Gene symbols that are underlined denote genes downregulated in the mammary gland of RC dams compared to WT dams.

### Gene Set Enrichment Analysis using Gene Ontology

To gain insight into the processes and pathways that could be regulated differentially between the different treatment groups, we performed Gene Set Enrichment Analysis (GSEA). Genes that showed a FDR ≤ 0.10 and had ENSEMBL annotations were tested against the background set of all expressed genes with ENSEMBL annotations. In this sense, for the enrichment analysis comparing the gene expression between KO *vs*. WT, 229 significant genes were tested against 13,334 background genes. Furthermore, for the enrichment analysis comparing RC *vs*. WT, 607 significant genes were evaluated against 12,772 background genes. The significant enrichment of Gene Ontology (GO) Biological Processes terms was tested using Fisher’s exact test. Briefly, the GO database defines functional classes of genes and assigns biological descriptors (GO terms) to genes on the basis of the properties of their encoded products. Genes that are assigned to the same GO term can be considered members of a category of genes that are more related in terms of some aspect of their biology than any given random sets of genes.

**Fig 2:**
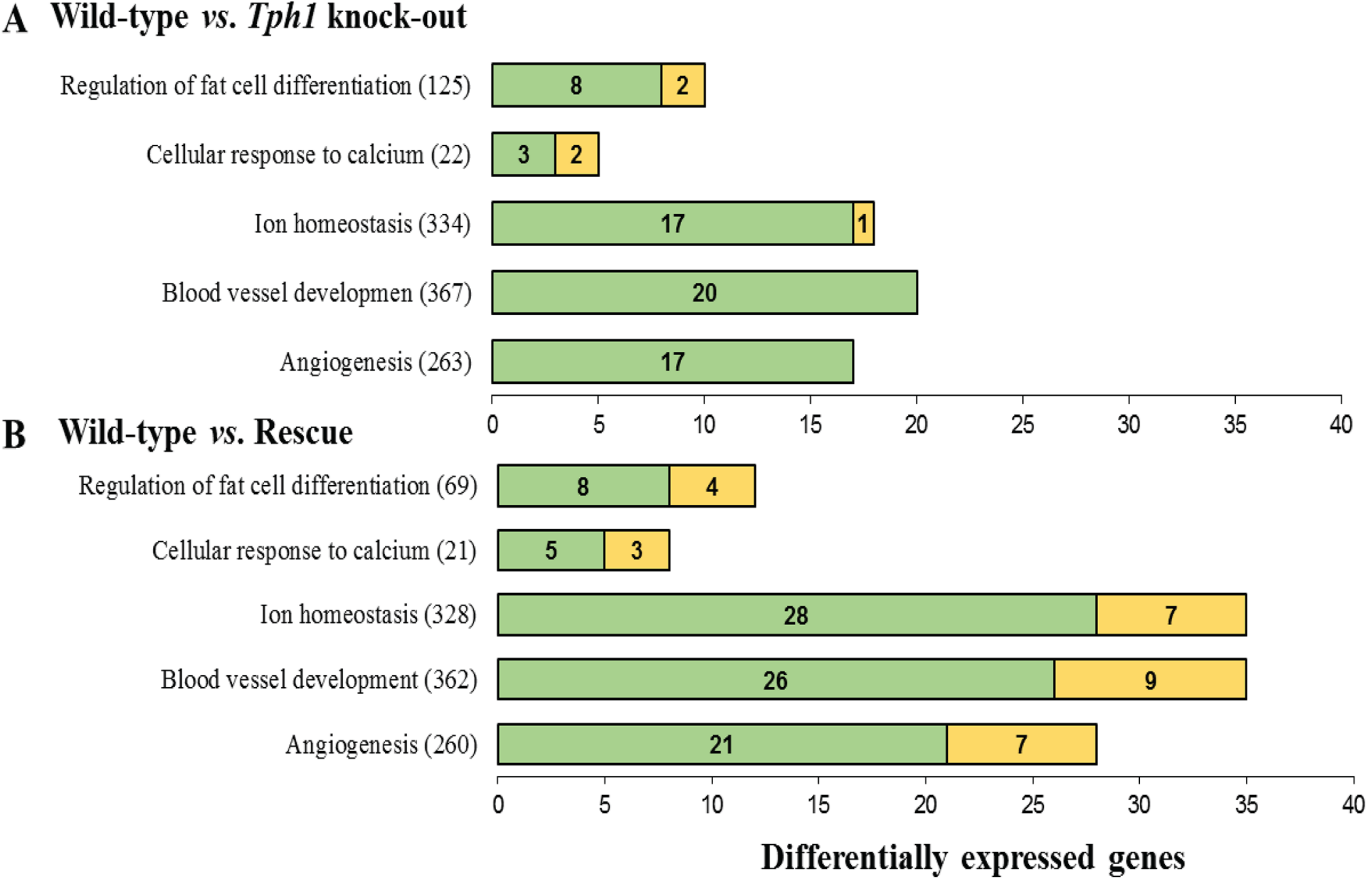
List of Gene Ontology (GO) biological process terms that were significantly enriched (FDR ≤ 0.01) with differentially expressed genes (DEG) detected simultaneously in both pairwise comparisons involving the wild-type (WT) mammary glands on day 10 of lactation: WT *vs*. Knock-out (A) and WT *vs*. Rescue (B). The graph shows the term name, the total number of genes within each term, and the number of genes up-regulated (green) and down-regulated (yellow) in the knock-out or rescue compared to the WT. Knock-out = Tryptophan hydroxylase (*Tph1*) deficient mice; Rescue = *Tph1* knock-out + 100 mg/kg daily injections of 5-hydroxytryptophan.

The GO categories (GO term names, number of genes within the term, total number of DEG) that showed significant enrichment with DEG detected in both WT *vs*. KO and WT *vs*. RC comparisons are displayed in Fig 2. For instance, the *angiogenesis* and *blood vessel development* functional terms were significantly enriched with DEG detected in the mammary gland of both KO and RC dams compared to WT dams. Interestingly, all of the significant genes in the KO (17 and 20, respectively) and the majority of the significant genes in the RC (21 and 26, respectively) were upregulated compared to the WT. A similar pattern of expression was observed for other shared functional categories: *ion homeostasis*, *cellular response to calcium*, and *regulation of fat cell differentiation*, where the majority of the DEG were upregulated in the mammary gland of KO and RC dams compared to the WT dams.

**Fig 3:**
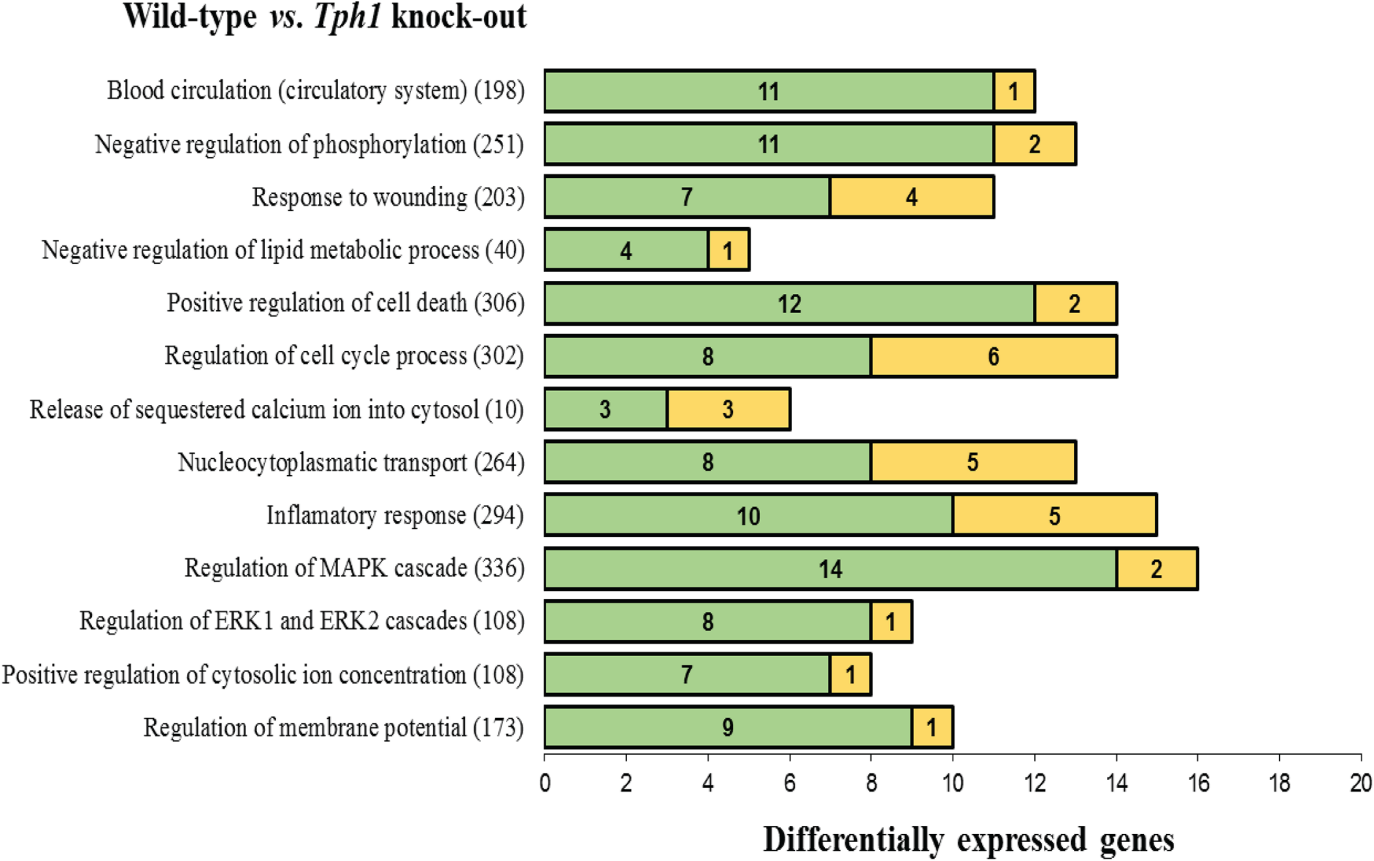
List of Gene Ontology (GO) terms that were significantly enriched (FDR ≤ 0.01) with differentially expressed genes (DEG) detected between wild-type (WT) *vs*. Knock-out mammary glands on day 10 of lactation. The graph shows the term name, the total number of genes within each term, and the number of genes up-regulated (green) and down-regulated (yellow) in the knock-out compared to the wild-type. Knock-out = Tryptophan hydroxylase (*Tph1*) deficient mice.

**Fig 4:**
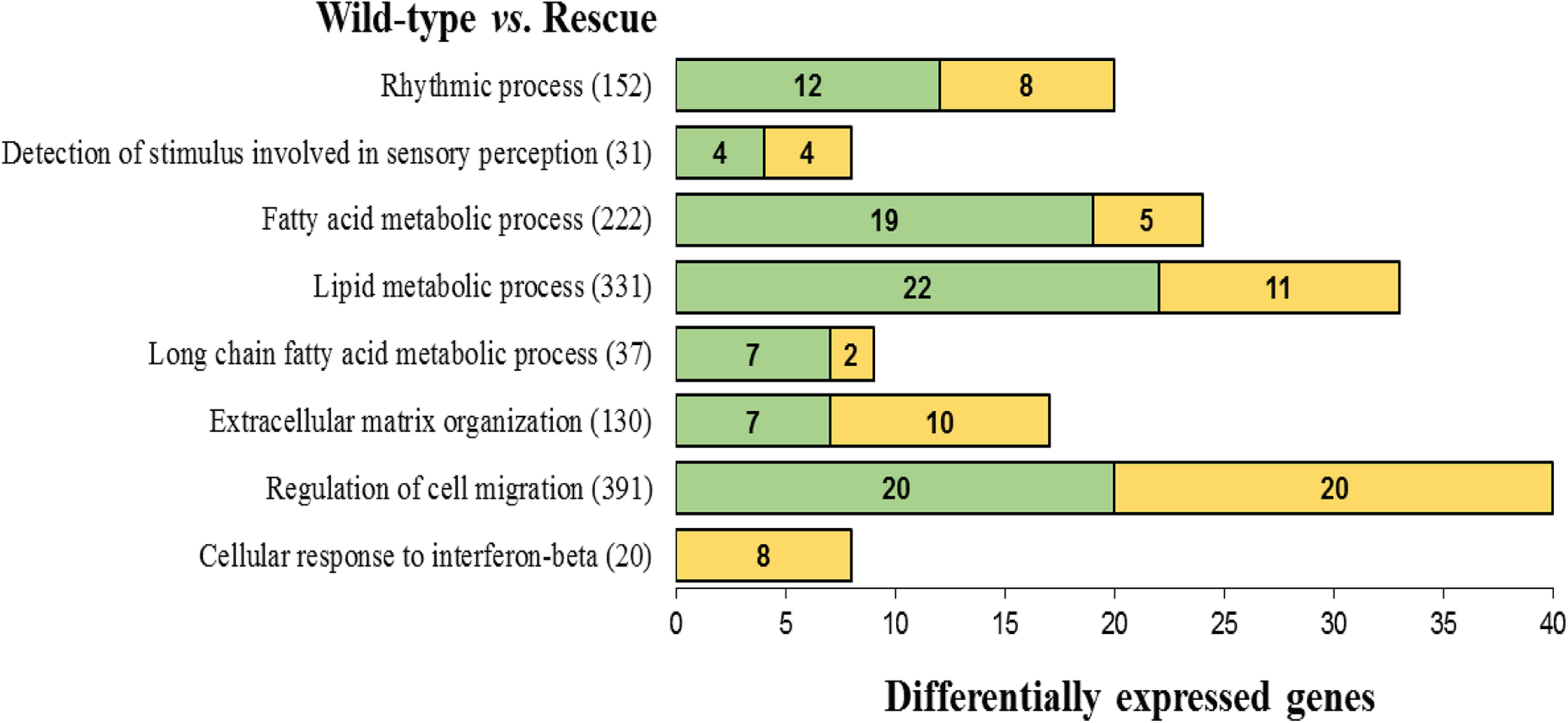
List of Gene Ontology (GO) terms that were significantly enriched (FDR ≤ 0.01) with differentially expressed genes (DEG) detected between wild-type (WT) *vs*. Rescue mammary gland on day 10 of lactation. The graph shows the term name, the total number of genes within each term, and the number of genes up-regulated (green) and down-regulated (yellow) in the Rescue compared to the wild-type. Rescue = Tryptophan hydroxylase (*Tph1*) knock-out + 100 mg/kg daily injections of 5-hydroxytryptophan.

The GO terms significantly enriched with DEG detected in the mammary gland between KO and WT dams are presented in Fig 3. Several relevant processes for the mammary gland and lactation were enriched such as those involved in intracellular signaling (e.g., ERK and MAP kinases cascades), ion regulation (e.g., *positive regulation of cytosolic ion concentration* and *release of calcium from the ER*), circulatory system, membrane potential, among others. Notably, the majority of the DEG within these functional terms were upregulated in the KO dams with the exception of two terms, namely *regulation of cell cycle process* and *nucleocytoplasmic transport*, in which roughly 40% of the DEG were upregulated and 60% were downregulated. The enriched GO terms with DEG detected between RC and WT mammary gland tissue are displayed in Fig 4. Several of the significant categories enriched after exogenous administration of 5-HTP are associated with lipid metabolism: lipid biosynthetic process, fatty acid and specifically long-chain fatty acid metabolic process. Cell migration and extracellular matrix organization, both dynamic and necessary processes in the mammary tissue for the purpose of lactation, were also enriched with DEG. All of the listed GO categories present variable percentages of genes that are up or downregulated within each term. For instance, all of the DEG for the *cellular response to interferon beta* GO term were downregulated whereas 60% of DEG for the *rhythmic processes* term and 50% of the DEG for the *cell migration* term were upregulated in mammary gland of RC dams compared to the WT dams.

### Gene Set Enrichment Analysis using Medical Subject Headings (MeSH)

The National Library of Medicine utilizes MeSH terms for indexing articles. In fact, each bibliographic reference is associated with a set of MeSH terms that describe the content of the publication, which is continually revised and updated. In contrast to GO where functional categories are mapped directly to genes, a unique feature of MeSH is that the terms are assigned to the literature. Therefore, because MeSH terms are associated with PubMed literature, an additional step of associating MeSH with genes is required.

The significant enrichment of MeSH terms with DEG was also analyzed using Fisher’s exact test (FDR ≤ 0.01). Presented in Table 4 are the list of selected and most relevant MeSH terms that were significantly enriched with DEG for the comparisons WT *vs*. KO and WT *vs*. RC. Only terms from the category *phenomena and processes* are displayed. The most significant MeSH terms are associated with cell proliferation, insulin resistance, and immune response, among others. The full list of significant MeSH terms for the WT *vs*. KO and WT vs RC pairwise comparisons can be found in S3 and S4 Files, respectively.

**Table 4:** Medical Subject Headings (MeSH) terms that were significantly enriched (FDR ≤ 0.01) with differentially expressed genes detected in the mammary gland on day 10 of lactation for the following pairwise comparisons: WT *vs*. KO and WT *vs*. RC.

MeSH Term name (MesH ID)	*P*-value
**Exclusive to WT *vs*. KO pairwise comparison**	
**Cell proliferation** (D049109)	1.6 x 10^−10^
**Insulin resistance** (D00733)	4.3 x 10^−10^
**Genetic predisposition to disease** (D020022)	1.2 x 10^−8^
**Liver regeneration** (D008115)	1.9 x 10^−8^
**Energy Metabolism** (D004734)	4.0 x 10^−8^
**Active transport, Cell nucleus** (D021581)	7.2 x 10^−8^
**Fasting** (D005215)	9.0 x 10^−8^
**Exclusive to WT *vs*. RC pairwise comparison**	
**Immunity, Innate** (D007113)	1.4 x 10^−11^
**Kinetics** (D007700)	1.4 x 10^−9^
**Bone density** (D015519)	1.9 x 10^−8^
**Cell movement** (D02465)	3.6 x 10^−8^
**Physiological feedback** (D025461)	4.0 x 10^−8^
**Cell communication** (D002450)	5.5 x 10^−8^
**Cell division** (D002455)	6.4 x 10^−8^
**Macrophage activation** (D008262)	9.3 x 10^−8^
**Cell death** (D016923)	1.5 x 10^−7^
**Neovascularization** (D018919)	3.5 x 10^−7^
**Obesity** (D009765)	6.5 x 10^−7^
**Permeability** (D010539)	6.5 x 10^−7^
**Enzyme activation** (D004789)	9.7 x 10^−7^

### Validation of overall gene expression

**Fig 5:**
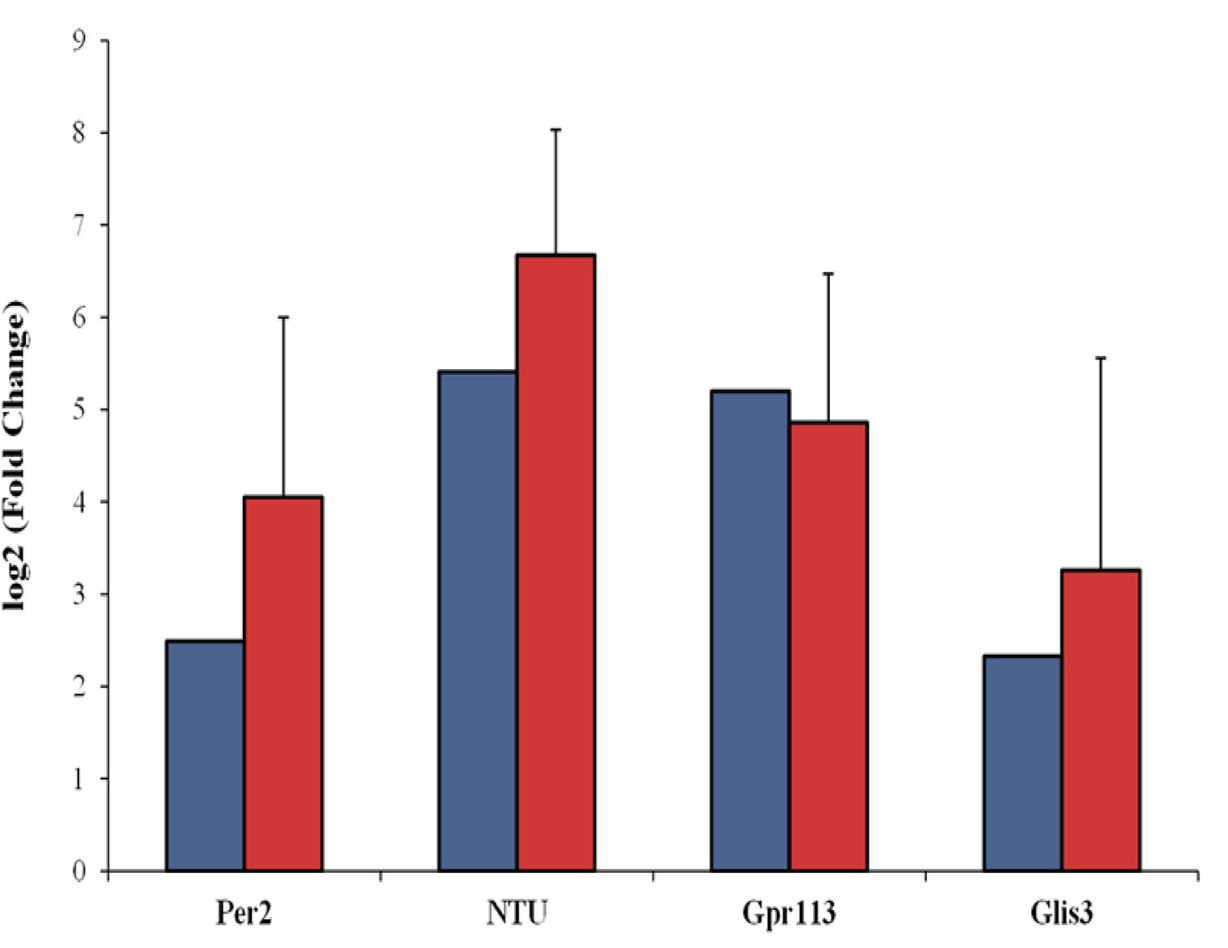
Validation of overall gene expression. Fold changes of four differentially expressed genes measured by RNA-Seq (blue) versus qRT-PCR (orange). Genes period circadian clock 2 (Per2), a novel transcript unit [Chr5:30,208,00730,212,059] (NTU), and G protein-coupled receptor 113 (Gpr113) were statistically upregulated in the mammary gland of RC dams while gene GLIS family zinc finger 3 (Glis3) was statistically upregulated in the mammary gland of WT dams.

To validate genes found to be significant in the RNA-Seq analysis, four differentially expressed genes, period circadian clock 2 (Per2), G protein-coupled receptor 113 (Gpr113), GLIS family zinc finger 3 (Glis3), and one novel transcript unit (NTU) were selected and their expression was assessed using by qPCR. The RNA-Seq analysis showed that Per2, Gpr113, and NTU were statistically upregulated in the mammary gland of RC dams while the gene Glis3 was statistically upregulated in the mammary gland of WT dams. Fig 5 displays the fold differences in gene expression measured by both RNA-Seq and qRT-PCR. The four genes displayed similar patterns of mRNA abundance with both methods.

## Discussion

Non-neuronal serotonin regulates a diverse set of physiological processes in numerous tissue types. Serotonin signals through approximately 15 different receptor subtypes, allowing for the regulation of multiple physiological events simultaneously [30]. Previous research has detailed the importance of serotonin in regulating mammary gland function and physiology [13,14,31,32,]. As such, serotonin has been described to regulate mammary gland cell turnover, milk protein gene expression, calcium signaling, and tight junction formation [9,10,13]. The presence of five serotonin receptors within the mammary gland [8] suggest the possibility that numerous other physiological functions may exist that have not yet been recognized. This study not only confirms serotonin participation in functions previously reported in the mammary gland, but also reveals physiological processes and functions, in which serotonin participates, that had not been reported previously and that might be critical for the process of lactation.

In this study, terms related to *calcium transport* and *cellular responses to calcium* were found among the most significant functional categories. We have previously demonstrated that serotonin is involved in calcium regulation, specifically in the transport of calcium into the mammary gland and milk during lactation [33]. In fact, we showed that the protein and gene expression of some of the major calcium transporters and pumps in the mammary gland were impacted by serotonin [9]. The upregulation of genes involved in the *release of sequestered calcium ion into the cytosol* exclusively in the serotonin deficient dams, might be reflecting the need for available calcium in those dams, given they are deficient in peripheral serotonin. In line with these findings, the enrichment of the MeSH term *bone density*, exclusively in the RC dams, provides further evidence of the presence of a mammary-bone serotonergic axis that functions to provide calcium for lactation [33].

The enrichment of pathways involved in aspects of *cell proliferation*, *cell cycle*, and *apoptosis*, is supported by previous research indicating that serotonin participates in mammary epithelial cell apoptosis and proliferation [9, 13]. The finding that genes involved in the *negative regulation of ERK*_*1/2*_ were exclusively upregulated in the mammary glands of serotonin deficient dams, compared to the wild-types, further confirms that serotonin regulates the expression of ERK_1/2_ [9]. The participation of serotonin in the regulation of ERK signaling cascade can have an impact in a large variety of processes in mammary epithelial cells which are essential for lactation, including *cell adhesion*, *cell migration*, *cell cycle*, *cell survival*, *differentiation*, *metabolism*, *proliferation*, among others. Interestingly, the majority of these functions were significantly altered in this study. Additionally, serotonin has previously been shown to be important for the assembly and disassembly of tight junctions [10, 11]. Our results demonstrate that the increased peripheral serotonin in the RC dams upregulates genes in the mammary gland that are involved in *tight junctions*, compared to wild-types dams. Therefore, this confirms that serotonin is involved in the regulation of a crucial function for milk synthesis and secretion in the mammary gland.

In conjunction with pathways that have previously been reported to be regulated by serotonin in the mammary gland, several new pathways were revealed in this study. Functional terms related to immune function were enriched in the mammary glands of serotonin deficient and the RC dams when compared to the wild-types dams. This finding is of interest for several reasons. It is well known that the developmental transition of the mammary gland to a non-secretory from a secretory state has a molecular signature that is very similar to an immune response [34]. It is plausible that serotonin signaling may have a role in mammary gland remodeling through regulation of an immune-like response. In fact, several functional categories related to immunity (i.e. *cytokine signaling*, *T-cell differentiation*, *IL-5 signaling pathway*, *interferon signaling*) were enriched exclusively in the rescue compared to the wild-type mammary glands. The finding that serotonin might be involved in mammary gland immune function opens up new possibilities for exploring the specific roles of this monoamine in mammary response to infection. Furthermore, serotonin was proposed to stimulate some immune markers in the mammary gland in response to high-fat diet feeding in rats [35] and also has been implicated in the activation of the immune responses and inflammation in several tissues and cell types throughout the body [36, 37, 38]. Here, the *inflammatory response* pathway was significantly impacted with the majority of the participating genes being upregulated in the serotonin deficient dams, compared to the wild-type dams. This finding might indicate a role of serotonin in the regulation of inflammatory responses in the mammary gland.

Another interesting finding is that the lack of peripheral serotonin significantly impacted *insulin signal* and *glucose metabolism* pathways in the mammary gland. In fact, the genes involved in these two functional categories were upregulated exclusively in the serotonin deficient dams, compared to the wild-types. It is known that serotonin can stimulate pancreatic β-cell proliferation, glucose and insulin secretion in both a pregnant mouse model and in a non-pregnant model, particularly during an insulin resistant state [39, 40]. In the mammary gland, we have determined that serotonin has effects on glucose transporters [33]. Serotonin participation in glucose metabolism and transport could potentially impact mammary gland lactose synthesis highlighting the importance of this monoamine for lactation. The role of serotonin in insulin signaling and its potential implications for mammary physiology and lactation is less clear and needs further research.

The enrichment of pathways related to lipid metabolism in the mammary gland of RC dams compared to wild-type dams could be beneficial for the assembly of milk fat, of which 50% occurs *de novo* in the mammary gland [41]. In addition, the upregulation of several genes involved in adipogenesis in the mammary gland of serotonin deficient dams might indicate that this monoamine plays a role regulating adipogenesis. Little work has focused on the effects of serotonin on adipogenesis, however, there is evidence that serotonin acts through the 5-HT2A receptor subtype to stimulate adipogenesis [42]. It has been shown that high fat diet consumption increased adipocytes in the mammary gland, decreasing milk protein gene expression and increasing serotonin production [35]. The involvement of serotonin in this pathway could be critical for mammary gland development. In line with this findings, MeSH enrichment analysis indicated that serotonin might also be involved in *obesity*. Interestingly, this term was enriched exclusively for the RC dams. Prepartal obesity delays the onset of lactogenesis stage II in humans, which is a critical barrier to successful breast-feeding outcomes [43]. This association between serotonin and obesity could open new avenues of research exploring the role of this monoamine in human lactation, as well as in the negative lactation outcomes of over-conditioned dairy cows.

The first physiological effect of serotonin described was its role in blood vessels as a vasoconstrictor [44]. Since then, it has been discovered that serotonin has a wide array of effects on the circulation depending on which receptor subtype serotonin interacts with [45,46,47]. Following this line, another important finding of this study is that blood circulation and angiogenic pathways were significantly affected in both the serotonin deficient and the rescue dams compared with WT. These two functions are particularly important for milk synthesis during lactation since they enable a more efficient transport of nutrients to the mammary epithelial cells and the removal of waste products. The functional categories *angiogenesis* and *blood vessels development* were altered in both the serotonin deficient and the rescue dams compared to wild-types, and the majority of the genes were upregulated. The functional category *blood circulation* (*circulatory system)* was also impacted but exclusively in the serotonin deficient dams compared to the wild-types. Further exploration of these pathways and the specific genes that are involved may shed light on the roles of serotonin in nutrient delivery to the mammary gland and its implications for lactation. It is known that serotonin stimulation of angiogenic pathways is common during breast cancer metastasis [48]. Regulation of this pathway by serotonin could prove to be important for the understanding of tumor progression.

A recent study revealed that the mammary gland adapts its clock during lactation [49]. These authors demonstrated that the mammary gland clock is attenuated during lactation in order to meet the demands of the nursing young at any point during the day [50]. This finding is important, because it demonstrates that peripheral clocks within tissues can act independently from the central clock within the suprachiasmatic nucleus. Serotonin is a precursor for melatonin synthesis within the pineal gland [51], and therefore it could be involved in the regulation of the central clock system. Interestingly we found that increased peripheral serotonin in the rescue dams upregulates genes associated to *rhythmic process* in the mammary gland during lactation. This finding could place peripheral serotonin as a possible regulator of a peripheral clock system within the mammary gland.

Thus far, our research on the involvement of serotonin in the mammary gland has only skimmed the surface of the potential effects. Our findings demonstrate a multiplicity of pathways being affected by serotonin in the mammary gland, more than had been originally indicated. It is important to note that we used a global *Tph1* deficient mouse model. Therefore, we cannot rule out the possibility that the observed effects on aspects related to calcium, glucose and insulin metabolism, and adipogenesis in the mammary tissue could be due to the overall effects of serotonin in other tissues such as the liver, the pancreas, the bone and adipocytes elsewhere. Whether the observed changes are due to a local/autocrine or a systemic action of serotonin in the mammary gland is still yet to be determined.

## Conclusion

Serotonin impacted numerous pathways which are relevant for mammary development, physiology and lactation. A more exhaustive examination of these pathways and the specific genes involved will provide evidence for new hypotheses involving serotonin function(s) in the mammary gland that might contribute to improving milk formation and lactation performance. The use of a mammary gland conditional *Tph1* knock-out mouse model will be imperative to accurately unravel the exclusive participation of serotonin in mammary physiology and lactation.

## Supporting Information

S1 File Full list of differentially expressed genes (FDR ≤ 0.01 and fold change ≥ 2) detected in the wild-type *vs*. *Tph1* knock-out pairwise comparison. [Tph1 = Tryptophan hydroxylase].
(TXT)

S2 File Full list of differentially expressed genes (FDR ≤ 0.01 and fold change ≥ 2) detected in the wild-type *vs*. Rescue pairwise comparison. [Rescue = Tryptophan hydroxylase (*Tph1*) knock-out + 100 mg/kg daily injections of 5-hydroxytryptophan].
(TXT)

S3 File Full list of significant Medical Subject Headings (MeSH) terms for the wild-type *vs*. *Tph1* (Tryptophan hydroxylase) knock-out pairwise comparison. (HTML)

S4 File Full list of significant Medical Subject Headings (MeSH) terms for the wild-type vs. Rescue pairwise comparison. [Rescue = Tryptophan hydroxylase (*Tph1*) knock-out + 100 mg/kg daily injections of 5-hydroxytryptophan].
(HTML)

S1 Table Primers used for the validation of gene expression. (DOCX)

S2 Table Summary of sequencing read alignments to the reference genome. (DOCX)
