# Supplementary material for: Transcriptomic Analysis of the Mouse Mammary Gland Reveals New Insights for the Role of Serotonin in Lactation
Source: PLoS One. 2015 Oct 15;10(10):e0140425. doi: 10.1371/journal.pone.0140425 (PMC4607441; doi:10.1371/journal.pone.0140425)
Supplement: S3 File — (HTML) [file pone.0140425.s003.html]

**Gene Set Enrichment Analysis (GSEA) using Medical Subject Headings (MeSH): Evaluation of differential gene expression between WT and KO**

Genome-wide gene expression in mammary gland samples was evaluated using RNA-Seq; these samples belong to wild-type dams (**WT**; n = 4) and Tryptophan hydroxylase (Tph1) knock-out dams (**KO**; i.e. Tph1 deficient; n = 4).

The significant enrichment of Medical Subject Headings terms (MeSH) with genes differentially expressed between WT and KO individuals was analyzed using Fisher’s exact test, a test of proportions based on the cumulative hypergeometric distribution.

Number of **[i] Significant genes (FDR < 0.10)** and **[ii] Background genes** used for this analysis: genes that showed a FDR < 0.10 and had ENSEMBL and MeSH annotations were tested against the background set of all genes analyzed with ENSEMBL and MeSH annotations.

```
## Significant Genes: 194  and Backgroung Genes: 11997
```

**Significant MeSH terms from the category [Phenomena and Processes]**:

List of the top 50 MeSH terms that were significantly enriched with differentially expressed genes. For each term, these results show [a] Mesh ID, [b] Mesh Name, and [c] the nominal P-value from the Fisher’s exact test (FDR < 0.01)

```
##  MeSH Term ID                            MeSH Term Name P-value
##       D015854                             Up-Regulation 9.7e-17
##       D002470                             Cell Survival 1.8e-15
##       D020935               MAP Kinase Signaling System 8.6e-15
##       D015536                           Down-Regulation 1.7e-14
##       D004305          Dose-Response Relationship, Drug 2.4e-14
##       D015533                Transcriptional Activation 1.0e-13
##       D014018                       Tissue Distribution 1.1e-13
##       D000375                                     Aging 1.3e-13
##       D006706                               Homeostasis 1.9e-13
##       D020218                         Response Elements 2.7e-13
##       D010805             Physical Conditioning, Animal 6.3e-13
##       D013997                              Time Factors 1.1e-12
##       D012038                              Regeneration 1.3e-12
##       D017930                           Genes, Reporter 1.5e-12
##       D017781                    Genes, Immediate-Early 1.8e-12
##       D013045                       Species Specificity 2.0e-12
##       D002452                                Cell Count 2.2e-12
##       D002454                      Cell Differentiation 2.9e-12
##       D049109                        Cell Proliferation 3.0e-12
##       D009024                             Morphogenesis 5.6e-12
##       D007333                        Insulin Resistance 9.3e-12
##       D015398                       Signal Transduction 9.6e-12
##       D017209                                 Apoptosis 1.3e-11
##       D015972    Gene Expression Regulation, Neoplastic 1.5e-11
##       D020013                         Calcium Signaling 1.7e-11
##       D009043                            Motor Activity 2.1e-11
##       D014162                              Transfection 3.7e-11
##       D008040                           Genetic Linkage 1.0e-10
##       D005314           Embryonic and Fetal Development 1.1e-10
##       D009119                        Muscle Contraction 1.1e-10
##       D002448                             Cell Adhesion 1.2e-10
##       D059467                             Transcriptome 1.2e-10
##       D015870                           Gene Expression 4.0e-10
##       D020022         Genetic Predisposition to Disease 4.3e-10
##       D001835                               Body Weight 6.0e-10
##       D008115                        Liver Regeneration 7.2e-10
##       D004742                Enhancer Elements, Genetic 9.2e-10
##       D024510                        Muscle Development 1.0e-09
##       D012150 Polymorphism, Restriction Fragment Length 1.2e-09
##       D013314                        Stress, Mechanical 1.3e-09
##       D004734                         Energy Metabolism 1.6e-09
##       D001665                             Binding Sites 2.7e-09
##       D005819                           Genetic Markers 2.9e-09
##       D021581            Active Transport, Cell Nucleus 3.2e-09
##       D015971   Gene Expression Regulation, Enzymologic 3.3e-09
##       D014945                             Wound Healing 3.9e-09
##       D012689           Sequence Homology, Nucleic Acid 4.0e-09
##       D017384                         Sequence Deletion 4.4e-09
##       D005215                                   Fasting 4.5e-09
##       D010766                           Phosphorylation 4.5e-09
```

**Significant MeSH terms from the category [Chemicals and Drugs]**:

List of the top 50 MeSH terms that were significantly enriched with differentially expressed genes. For each term, these results show [a] Mesh ID, [b] Mesh Name, and [c] the nominal P-value from the Fisher’s exact test (FDR < 0.01)

```
##  MeSH Term ID                               MeSH Term Name P-value
##       D009124                              Muscle Proteins 3.9e-21
##       D008156                                  Luciferases 5.4e-17
##       D015415                           Biological Markers 9.0e-16
##       D007328                                      Insulin 1.3e-15
##       D002118                                      Calcium 2.0e-14
##       D020033                             Protein Isoforms 4.0e-14
##       D017570                                 MyoD Protein 2.1e-13
##       D048049   Extracellular Signal-Regulated MAP Kinases 2.1e-13
##       D050778                  NFATC Transcription Factors 4.3e-13
##       D019703                                  Calcineurin 4.7e-13
##       D017362  Cyclic AMP Response Element-Binding Protein 1.6e-12
##       D053773             Transforming Growth Factor beta1 2.6e-12
##       D016755                Proto-Oncogene Proteins c-jun 2.9e-12
##       D019938                                    Cyclin D1 4.1e-12
##       D008070                          Lipopolysaccharides 5.3e-12
##       D051057                Proto-Oncogene Proteins c-akt 5.6e-12
##       D020780                   Matrix Metalloproteinase 9 6.1e-12
##       D015534                             Trans-Activators 6.1e-12
##       D000242                                   Cyclic AMP 6.3e-12
##       D007334                 Insulin-Like Growth Factor I 1.5e-11
##       D006133                            Growth Substances 3.5e-11
##       D000209                         Acute-Phase Proteins 5.1e-11
##       D064326                   MEF2 Transcription Factors 5.2e-11
##       D016207                                    Cytokines 5.9e-11
##       D000906                                   Antibodies 8.5e-11
##       D051792 Basic Helix-Loop-Helix Transcription Factors 1.1e-10
##       D016760                Proto-Oncogene Proteins c-fos 1.1e-10
##       D017874                     Immediate-Early Proteins 1.5e-10
##       D047428                    Protein Kinase Inhibitors 1.7e-10
##       D007527                                   Isoenzymes 2.8e-10
##       D005947                                      Glucose 3.4e-10
##       D051176                                 beta Catenin 3.9e-10
##       D018690               Excitatory Amino Acid Agonists 4.0e-10
##       D009419                        Nerve Tissue Proteins 4.9e-10
##       D020928            Mitogen-Activated Protein Kinases 5.0e-10
##       D051795    Hypoxia-Inducible Factor 1, alpha Subunit 5.5e-10
##       D010982               Platelet-Derived Growth Factor 5.8e-10
##       D051766              Early Growth Response Protein 1 6.3e-10
##       D018836                       Inflammation Mediators 6.3e-10
##       D019950           Mitogen-Activated Protein Kinase 1 9.8e-10
##       D026362                        Serum Response Factor 9.8e-10
##       D011518                      Proto-Oncogene Proteins 1.1e-09
##       D011494                              Protein Kinases 1.2e-09
##       D011993                  Recombinant Fusion Proteins 1.3e-09
##       D003907                                Dexamethasone 1.3e-09
##       D000199                                       Actins 1.4e-09
##       D051858               Forkhead Transcription Factors 1.9e-09
##       D018808                    Transcription Factor AP-1 1.9e-09
##       D015703                                 Antigens, CD 1.9e-09
##       D019869               Phosphatidylinositol 3-Kinases 1.9e-09
```

**Significant MeSH terms from the category [Diseases]**:

List of the top 50 MeSH terms that were significantly enriched with differentially expressed genes. For each term, these results show [a] Mesh ID, [b] Mesh Name, and [c] the nominal P-value from the Fisher’s exact test (FDR < 0.01)

```
##  MeSH Term ID                              MeSH Term Name P-value
##       D004195                      Disease Models, Animal 1.1e-15
##       D018450                         Disease Progression 3.5e-12
##       D002471             Cell Transformation, Neoplastic 5.6e-12
##       D007333                          Insulin Resistance 9.3e-12
##       D007249                                Inflammation 3.5e-11
##       D005355                                    Fibrosis 9.7e-11
##       D006330                   Heart Defects, Congenital 2.3e-10
##       D000860                                      Anoxia 3.2e-10
##       D006332                                Cardiomegaly 3.2e-10
##       D020022           Genetic Predisposition to Disease 4.3e-10
##       D001835                                 Body Weight 6.0e-10
##       D006333                               Heart Failure 4.0e-09
##       D009362                         Neoplasm Metastasis 4.4e-09
##       D006984                                 Hypertrophy 4.7e-09
##       D000208                               Acute Disease 8.3e-09
##       D002872                         Chromosome Deletion 2.4e-08
##       D006965                                 Hyperplasia 5.9e-08
##       D008103                             Liver Cirrhosis 1.7e-07
##       D002908                             Chronic Disease 1.8e-07
##       D005234                                 Fatty Liver 2.1e-07
##       D004681 Encephalomyelitis, Autoimmune, Experimental 2.9e-07
##       D008113                             Liver Neoplasms 3.2e-07
##       D050197                             Atherosclerosis 4.5e-07
##       D009133                            Muscular Atrophy 5.6e-07
##       D009361                       Neoplasm Invasiveness 7.5e-07
##       D005334                                       Fever 8.1e-07
##       D009389              Neovascularization, Pathologic 8.8e-07
##       D001284                                     Atrophy 8.9e-07
##       D019446                                 Endotoxemia 9.3e-07
##       D020244          Infarction, Middle Cerebral Artery 9.4e-07
##       D006528                   Carcinoma, Hepatocellular 9.9e-07
##       D001161                            Arteriosclerosis 1.1e-06
##       D003920                           Diabetes Mellitus 1.5e-06
##       D001249                                      Asthma 1.6e-06
##       D011014                                   Pneumonia 1.6e-06
##       D003922                   Diabetes Mellitus, Type 1 2.3e-06
##       D009202                            Cardiomyopathies 2.5e-06
##       D009203                       Myocardial Infarction 2.8e-06
##       D015427                          Reperfusion Injury 3.1e-06
##       D004487                                       Edema 3.5e-06
##       D003921             Diabetes Mellitus, Experimental 3.6e-06
##       D012162                        Retinal Degeneration 3.7e-06
##       D009765                                     Obesity 3.8e-06
##       D012640                                    Seizures 4.5e-06
##       D056486                   Drug-Induced Liver Injury 5.5e-06
##       D000544                           Alzheimer Disease 6.0e-06
##       D011125                  Adenomatous Polyposis Coli 6.5e-06
##       D006967                            Hypersensitivity 6.5e-06
##       D012871                               Skin Diseases 7.6e-06
##       D001930                              Brain Injuries 8.1e-06
```
