# Supplementary material for: Transcriptomic Analysis of the Mouse Mammary Gland Reveals New Insights for the Role of Serotonin in Lactation
Source: PLoS One. 2015 Oct 15;10(10):e0140425. doi: 10.1371/journal.pone.0140425 (PMC4607441; doi:10.1371/journal.pone.0140425)
Supplement: S4 File — [Rescue = Tryptophan hydroxylase (Tph1) knock-out + 100 mg/kg daily injections of 5-hydroxytryptophan]. (HTML) [file pone.0140425.s004.html]

**Gene Set Enrichment Analysis (GSEA) using Medical Subject Headings (MeSH): Evaluation of differential gene expression between WT and RC**

Genome-wide gene expression in mammary gland samples was evaluated using RNA-Seq; these samples belong to wild-type dams (**WT**; n = 4) and Tryptophan hydroxylase (Tph1) deficient dams (knock-down) that were injected daily with 5-HTP (**RC**; n = 4).

The significant enrichment of Medical Subject Headings terms (MeSH) with genes differentially expressed between WT and RC individuals was analyzed using Fisher’s exact test, a test of proportions based on the cumulative hypergeometric distribution.

Number of **[i] Significant genes (FDR < 0.10)** and **[ii] Background genes** used for this analysis: genes that showed a FDR < 0.10 and had ENSEMBL and MeSH annotations were tested against the background set of all genes analyzed with ENSEMBL and MeSH annotations.

```
## Significant Genes: 574  and Backgroung Genes: 11434
```

**Significant MeSH terms from the category [Phenomena and Processes]**:

List of the top 50 MeSH terms that were significantly enriched with differentially expressed genes. For each term, these results show [a] Mesh ID, [b] Mesh Name, and [c] the nominal P-value from the Fisher’s exact test (FDR < 0.01)

```
##  MeSH Term ID                          MeSH Term Name P-value
##       D002454                    Cell Differentiation 1.1e-17
##       D015854                           Up-Regulation 7.4e-17
##       D005810                        Multigene Family 6.2e-16
##       D005865                         Gestational Age 1.4e-14
##       D001835                             Body Weight 2.0e-14
##       D014162                            Transfection 2.3e-14
##       D007113                        Immunity, Innate 8.8e-14
##       D002448                           Cell Adhesion 1.0e-13
##       D012038                            Regeneration 1.3e-13
##       D004305        Dose-Response Relationship, Drug 1.4e-13
##       D006706                             Homeostasis 2.6e-13
##       D000375                                   Aging 4.0e-13
##       D014018                     Tissue Distribution 1.0e-12
##       D002470                           Cell Survival 1.3e-12
##       D015398                     Signal Transduction 1.7e-12
##       D015536                         Down-Regulation 1.7e-12
##       D005314         Embryonic and Fetal Development 3.0e-12
##       D015870                         Gene Expression 3.5e-12
##       D020935             MAP Kinase Signaling System 5.3e-12
##       D013997                            Time Factors 8.4e-12
##       D020013                       Calcium Signaling 1.0e-11
##       D015972  Gene Expression Regulation, Neoplastic 1.4e-11
##       D007700                                Kinetics 2.8e-11
##       D010766                         Phosphorylation 3.4e-11
##       D015971 Gene Expression Regulation, Enzymologic 3.5e-11
##       D015533              Transcriptional Activation 4.1e-11
##       D017930                         Genes, Reporter 6.0e-11
##       D017209                               Apoptosis 6.8e-11
##       D020218                       Response Elements 7.8e-11
##       D004032                                    Diet 9.0e-11
##       D004742              Enhancer Elements, Genetic 1.5e-10
##       D002452                              Cell Count 2.1e-10
##       D015519                            Bone Density 5.5e-10
##       D002465                           Cell Movement 1.1e-09
##       D025461                 Feedback, Physiological 1.3e-09
##       D014945                           Wound Healing 1.3e-09
##       D002450                      Cell Communication 1.8e-09
##       D002455                           Cell Division 2.1e-09
##       D009024                           Morphogenesis 2.5e-09
##       D008262                   Macrophage Activation 3.3e-09
##       D016923                              Cell Death 5.5e-09
##       D008040                         Genetic Linkage 6.9e-09
##       D018919         Neovascularization, Physiologic 1.3e-08
##       D055495                            Neurogenesis 2.3e-08
##       D009765                                 Obesity 2.6e-08
##       D010539                            Permeability 2.6e-08
##       D006720                              Homozygote 3.3e-08
##       D004789                       Enzyme Activation 4.1e-08
##       D013045                     Species Specificity 4.5e-08
##       D001665                           Binding Sites 4.7e-08
```

**Significant MeSH terms from the category [Chemicals and Drugs]**:

List of the top 50 MeSH terms that were significantly enriched with differentially expressed genes. For each term, these results show [a] Mesh ID, [b] Mesh Name, and [c] the nominal P-value from the Fisher’s exact test (FDR < 0.01)

```
##  MeSH Term ID                               MeSH Term Name P-value
##       D016207                                    Cytokines 6.3e-14
##       D003907                                Dexamethasone 2.5e-13
##       D051792 Basic Helix-Loop-Helix Transcription Factors 3.8e-13
##       D015415                           Biological Markers 4.5e-13
##       D015703                                 Antigens, CD 1.7e-12
##       D051057                Proto-Oncogene Proteins c-akt 2.6e-12
##       D008024                                      Ligands 4.3e-12
##       D020033                             Protein Isoforms 7.4e-12
##       D008070                          Lipopolysaccharides 1.3e-11
##       D000242                                   Cyclic AMP 1.4e-11
##       D018398                         Homeodomain Proteins 2.5e-11
##       D006133                            Growth Substances 2.7e-11
##       D005938                              Glucocorticoids 3.2e-11
##       D050778                  NFATC Transcription Factors 3.2e-11
##       D014409                  Tumor Necrosis Factor-alpha 3.2e-11
##       D018836                       Inflammation Mediators 5.9e-11
##       D002135                     Calcium-Binding Proteins 7.6e-11
##       D005947                                      Glucose 1.1e-10
##       D007371                             Interferon-gamma 1.2e-10
##       D051858               Forkhead Transcription Factors 1.3e-10
##       D000906                                   Antibodies 1.5e-10
##       D051176                                 beta Catenin 2.8e-10
##       D011994                         Recombinant Proteins 3.2e-10
##       D007334                 Insulin-Like Growth Factor I 3.8e-10
##       D019869               Phosphatidylinositol 3-Kinases 4.5e-10
##       D048052           Mitogen-Activated Protein Kinase 3 5.3e-10
##       D006023                                Glycoproteins 5.7e-10
##       D016222                   Fibroblast Growth Factor 2 5.8e-10
##       D013755                 Tetradecanoylphorbol Acetate 6.0e-10
##       D015347                                   RNA Probes 6.7e-10
##       D009124                              Muscle Proteins 1.1e-09
##       D007328                                      Insulin 1.1e-09
##       D051059                        PTEN Phosphohydrolase 1.9e-09
##       D004791                            Enzyme Inhibitors 2.0e-09
##       D016755                Proto-Oncogene Proteins c-jun 2.1e-09
##       D025521                    Tumor Suppressor Proteins 2.5e-09
##       D002118                                      Calcium 2.5e-09
##       D020778                   Matrix Metalloproteinase 2 2.7e-09
##       D014157                        Transcription Factors 3.0e-09
##       D010455                                     Peptides 3.6e-09
##       D011518                      Proto-Oncogene Proteins 3.9e-09
##       D008156                                  Luciferases 5.4e-09
##       D005346                    Fibroblast Growth Factors 6.8e-09
##       D060850                     LIM-Homeodomain Proteins 7.6e-09
##       D015820                                    Cadherins 8.0e-09
##       D008562                       Membrane Glycoproteins 8.1e-09
##       D051153                                 Wnt Proteins 8.2e-09
##       D011993                  Recombinant Fusion Proteins 1.3e-08
##       D051798       Inhibitor of Differentiation Protein 1 1.8e-08
##       D003094                                     Collagen 1.8e-08
```

**Significant MeSH terms from the category [Diseases]**:

List of the top 50 MeSH terms that were significantly enriched with differentially expressed genes. For each term, these results show [a] Mesh ID, [b] Mesh Name, and [c] the nominal P-value from the Fisher’s exact test (FDR < 0.01)

```
##  MeSH Term ID                              MeSH Term Name P-value
##       D004195                      Disease Models, Animal 4.7e-17
##       D007249                                Inflammation 8.1e-15
##       D001835                                 Body Weight 2.0e-14
##       D008103                             Liver Cirrhosis 1.3e-10
##       D005355                                    Fibrosis 4.5e-10
##       D050197                             Atherosclerosis 9.4e-10
##       D002471             Cell Transformation, Neoplastic 3.3e-09
##       D001249                                      Asthma 1.2e-08
##       D009765                                     Obesity 2.6e-08
##       D002908                             Chronic Disease 6.3e-08
##       D003924                   Diabetes Mellitus, Type 2 7.4e-08
##       D004681 Encephalomyelitis, Autoimmune, Experimental 7.9e-08
##       D009203                       Myocardial Infarction 8.2e-08
##       D016535                   Bronchial Hyperreactivity 1.2e-07
##       D018805                                      Sepsis 2.1e-07
##       D006333                               Heart Failure 2.2e-07
##       D005234                                 Fatty Liver 2.6e-07
##       D014376                                Tuberculosis 4.1e-07
##       D009389              Neovascularization, Pathologic 4.4e-07
##       D013203                   Staphylococcal Infections 4.6e-07
##       D007333                          Insulin Resistance 4.6e-07
##       D020022           Genetic Predisposition to Disease 5.0e-07
##       D000208                               Acute Disease 7.0e-07
##       D006470                                  Hemorrhage 9.0e-07
##       D018450                         Disease Progression 9.2e-07
##       D003092                                     Colitis 1.2e-06
##       D012640                                    Seizures 1.4e-06
##       D008171                               Lung Diseases 1.4e-06
##       D015427                          Reperfusion Injury 1.7e-06
##       D009374                     Neoplasms, Experimental 1.9e-06
##       D012878                              Skin Neoplasms 2.0e-06
##       D001161                            Arteriosclerosis 2.2e-06
##       D008088                                 Listeriosis 2.2e-06
##       D009362                         Neoplasm Metastasis 2.6e-06
##       D012481               Salmonella Infections, Animal 2.8e-06
##       D006330                   Heart Defects, Congenital 3.1e-06
##       D020256                Choroidal Neovascularization 4.1e-06
##       D007674                             Kidney Diseases 4.1e-06
##       D003110                           Colonic Neoplasms 4.6e-06
##       D008546                      Melanoma, Experimental 6.0e-06
##       D012164                            Retinal Diseases 6.5e-06
##       D011014                                   Pneumonia 8.0e-06
##       D008113                             Liver Neoplasms 1.1e-05
##       D009336                                    Necrosis 1.1e-05
##       D001168                                   Arthritis 1.2e-05
##       D018487               Ventricular Dysfunction, Left 1.2e-05
##       D006965                                 Hyperplasia 1.2e-05
##       D011658                          Pulmonary Fibrosis 1.6e-05
##       D002294                    Carcinoma, Squamous Cell 1.6e-05
##       D006332                                Cardiomegaly 1.7e-05
```
