# Supplementary material for: Transcriptomic Analysis of the Mouse Mammary Gland Reveals New Insights for the Role of Serotonin in Lactation
Source: PLoS One. 2015 Oct 15;10(10):e0140425. doi: 10.1371/journal.pone.0140425 (PMC4607441; doi:10.1371/journal.pone.0140425)
Supplement: S1 Table — (DOCX) [file pone.0140425.s005.docx]

**S1 Table.** Primers used for the validation of gene expression

| **Gene^1^** | **Primer sequence (5’🡪3’)** | **Chromosome location**  **Exon-exon junction** | **Amplicon (bp)** |
| --- | --- | --- | --- |
| Per2 | **F:** GTGCCTGGAACACAGGTAGG  **R:** CTCGGGACTCTTCTGAGAGC | Chr. 1, exons 4-5 | 171 |
| NTU | **F:** TGGGAGAGGGACATTTAGGG  **R:** CGAACTTCTGATCCCTCTGC | Chr. 5, exons 1-2 | 157 |
| Gpr113 | **F:** CGGAACAGCTAGCACAAAGC  **R:** CTGTCCATCTCCTGTACTGC | Chr. 5, exons 4-5 | 153 |
| Glis3 | **F:** AGGCAGTCCTCAAAGGAAGG  **R:** GCCAAATGGTATCCATGTCC | Chr. 19, exons 2-3 | 131 |
| Rsp15 | **F:** TTGAGAAAGGCCAAAAAGGA  **R:** GTTGAAGGTCTTGCCGTTGT | Chr 10, exons 3-4 | 131 |

**^1^** Per2: period circadian clock 2; NTU: novel transcript unit [Chr5:30,208,007-30,212,059]; Gpr113: G protein-coupled receptor 113; GLIS: family zinc finger 3. F = forward and R= reverse; bp = base pairs.
