# Supplementary material for: Transcriptomic Analysis of the Mouse Mammary Gland Reveals New Insights for the Role of Serotonin in Lactation
Source: PLoS One. 2015 Oct 15;10(10):e0140425. doi: 10.1371/journal.pone.0140425 (PMC4607441; doi:10.1371/journal.pone.0140425)
Supplement: S2 Table — (DOCX) [file pone.0140425.s006.docx]

**S2 Table.** Summary of sequencing read alignments to the reference genome

| Sample | | Total  Reads | Total Mapped Reads | Percent Mapped | Uniquely mapped Reads | Percent uniquely mapped | Reads mapped to annotated exons |
| --- | --- | --- | --- | --- | --- | --- | --- |
| WT_1_ | 21,143,325 | | 16,758,885 | 79.3 | 16,334,929 | 97.5 | 15,801,052 |
| WT_2_ | 40,107,749 | | 31,748,474 | 79.2 | 31,001,695 | 97.6 | 30,107,528 |
| WT_3_ | 37,924,110 | | 30,397,201 | 80.2 | 29,650,039 | 97.5 | 27,677,177 |
| WT_4_ | 37,400,685 | | 29,606,473 | 79.2 | 28,858,916 | 97.4 | 27,419,277 |
|  |  | |  |  |  |  |  |
| RC_1_ | 41,879,514 | | 33,522,345 | 80.0 | 32,691,532 | 97.5 | 31,697,780 |
| RC_2_ | 14,894,887 | | 11,659,265 | 78.3 | 11,342,481 | 97.3 | 10,941,514 |
| RC_3_ | 11,332,903 | | 8,475,976 | 74.8 | 8,292,462 | 97.8 | 8,017,634 |
| RC_4_ | 50,781,977 | | 41,396,292 | 81.5 | 40,332,400 | 97.4 | 38,511,245 |
|  |  | |  |  |  |  |  |
| KO_1_ | 22,438,327 | | 17,149,383 | 76.4 | 16,734,908 | 97.6 | 15,996,717 |
| KO_2_ | 18,184,796 | | 14,008,906 | 77.0 | 13,697,721 | 97.8 | 13,253,885 |
| KO_3_ | 40,274,776 | | 32,175,261 | 79.9 | 31,415,726 | 97.6 | 30,450,482 |
| KO_4_ | 19,867605 | | 15,691,953 | 79.0 | 15,341,582 | 97.8 | 14,519,108 |

Samples belong to wild-type dams (WT), Tryptophan hydroxylase (*Tph1*) knock-out dams (*Tph1* deficient; KO), and *Tph1* deficient dams injected daily with 5-HTP (RC).
